# Differential peptide-dependent regulation of growth hormone (GH): A comparative analysis in pituitary cultures of reptiles, birds, and mammals

**DOI:** 10.1016/j.heliyon.2024.e33060

**Published:** 2024-06-14

**Authors:** Valeria A. Urban-Sosa, José Ávila-Mendoza, Martha Carranza, Carlos G. Martínez-Moreno, Maricela Luna, Carlos Arámburo

**Affiliations:** Departamento de Neurobiología Celular y Molecular, Instituto de Neurobiología, Campus Juriquilla, Universidad Nacional Autónoma de México, Querétaro, Qro., 76230, Mexico

**Keywords:** Growth hormone, Pituitary, GH-Regulatory peptides, Pit-1, Iguana

## Abstract

Growth hormone (GH) is a pituitary protein that exerts pleiotropic roles in vertebrates. The mechanisms regulating GH synthesis and secretion are finely controlled by hypothalamic neuropeptides and other factors. These processes have been considerably studied in mammals but are still poorly understood in other groups. To better understand the pituitary GH regulation during vertebrate phylogeny, we compared the effects of incubating several peptides on cultures of ex-vivo pituitary fragments obtained from representative specimens of reptiles (iguana), birds (chicken) and mammals (rat). The peptides used were: growth hormone-releasing hormone (GHRH), thyrotropin-releasing hormone (TRH), pituitary adenylate cyclase-activating polypeptide (PACAP), ghrelin, gonadotropin-releasing hormone (GnRH), and somatostatin (SST). In rat pituitary cultures, GH secretion was stimulated by GHRH and TRH, while *gh* mRNA expression was increased by GHRH and PACAP. In the case of chicken pituitaries, GH release was promoted by GHRH, ghrelin, PACAP, and GnRH, although the latter two had a dual effect since at a shorter incubation time they decreased GH secretion; in turn, *gh* mRNA expression was significantly stimulated by TRH, PACAP, and GnRH. The most intense effects were observed in iguana pituitary cultures, where GH secretion was significantly augmented by GHRH, PACAP, TRH, ghrelin, and GnRH; while *gh* mRNA expression was stimulated by GHRH, TRH, and PACAP, but inhibited by ghrelin and SST. Also, in the three species, SST was able to block the GHRH-stimulated GH release. Furthermore, it was found that the expression of *Pou1f1* mRNA was increased with greater potency by GHRH and PACAP in the iguana, than in chicken or rat pituitary cultures. Additionally, *in-silico* analysis of the *gh* gene promoter structures in the three species showed that the reptilian promoter has more Pit-1 consensus binding sites than their avian and mammalian counterparts. Taken together, results demonstrate that pituitary peptide-mediated GH regulatory mechanisms are differentially controlled along vertebrate evolution.

## Highligths

• Peptide-mediated GGH regulation was studied in reptiles, birds and mammals.
• GH regulation mechanisms show differential changes during vertebrate evolution.
• In reptiles, more peptides participate in GH regulation, as compared to mammals.
• *Gh* gene promoter has more PIT1 binding sites in iguana than in chicken and rat.

## 1 Introduction

Pituitary growth hormone (GH) plays an important role in the control of somatic growth and development, since it is involved in the regulation of cell proliferation and differentiation, as well as in the modulation of several metabolic processes in various tissues [1], and these effects are conserved in all vertebrates [2]. GH also exerts pleiotropic actions in many organs along vertebrate groups [3]. Several tissues are targets of GH activities, including those related with somatic growth (muscle, bone, and adipose tissue) [4], but also others where GH has tissue-specific roles. It has been shown that GH acts as a neurotrophic factor in the nervous system of fish [5], reptiles [6], birds [7], and mammals [8]. Also, GH is involved in modulating several aspects of the immune response in birds [9] and mammals [10]. Likewise, it has been described that GH has important actions on both the female and male reproductive systems ([[11], [12], [13]]). Some of these actions are conserved among vertebrates, although there are also species-specific actions; for example, GH is involved in the acclimatization to saltwater in teleosts ([[14], [15], [16]]). Furthermore, it has been previously described in fish and in the green iguana (an ectothermic reptilian species belonging to the Squamata order) that circulating levels of GH significantly increased when animals were exposed to changes in environmental temperature, likely as an adaptive mechanism ([[17], [18], [19]]). This diversity in GH actions could be related to the complex mechanisms involved in the regulation of its synthesis and secretion in the pituitary somatotrophs, which are essentially controlled by several hypothalamic neuropeptides and other factors ([[20], [21], [22]]).

In mammals, the main positive regulator of GH is the growth hormone releasing hormone (GHRH), while somatostatin (SST) is its main negative controller [23], and these stimulatory and inhibitory functions, respectively, are well conserved in other vertebrate groups, such as in fish ([24,25]), amphibians [26], reptiles ([27,28]), and birds ([29,30]). Once GH is released from the pituitary, it binds to its specific receptor (GHR) in several target tissues, but particularly in the liver, and stimulates the synthesis and secretion of insulin-like growth factor 1 (IGF-1) which, in turn, exerts negative feedback on GH expression and release [31]. On the other hand, it has been shown that several other hypothalamic hypophysiotropic neuropeptides are involved in GH regulation in vertebrates [21], including the pituitary adenylate cyclase-activating polypeptide (PACAP) [32], thyrotropin releasing hormone (TRH) [33]; and gonadotropin releasing hormone (GnRH) [34]. Thus, PACAP has been described as a stimulator of GH secretion in fish [35], amphibians [36], and birds [37], while in mammals it has shown differential responses, being stimulatory in rats [38] and pigs [39] but without effect or even inhibitory in sheep [40]. Likewise, TRH modulates the release of GH in fish [41], amphibia [42], reptiles [27], birds [43], and mammals [44]. Also, the ability of GnRH to act upon GH secretion has been described in fish [34] and mammals [45]. Furthermore, ghrelin -a gastropeptide produced mainly in the stomach in response to fasting- has also been shown to strongly induce pituitary GH release [46] in fish [47], amphibia [48], reptiles [49], birds [50], and mammals [51]. Although the primary function of these peptides has been associated to the regulation of other pituitary hormones, several studies indicate that under certain physiological conditions and in different species they may play important roles as GH-regulatory peptides [52].

To regulate GH synthesis, peptides bind to specific G-protein coupled receptors to stimulate or inhibit intracellular signaling pathways. In general, by regulating the cAMP/PKA/CREB signaling pathway activity, peptides induce the expression of the transcription factor Pit-1, which in turn induces the *gh* mRNA expression [21]. Moreover, the regulation of GH secretion involves additional mechanisms, including inositol trisphosphate (IP3), calcium mobilization, activation of Na^+^ and K^+^ channels, protein kinase C (PKC), among others, to control GH exocytosis from the secretory granules [21].

We previously described the primary structure and distribution of GH, GHR, GHRH, PACAP, TRH, SST, and IGF-1 in the green iguana ([53,54]). On the other hand, a significant increase in the *gh* mRNA expression and GH release was observed when these reptiles were exposed to low environmental temperatures, an effect where other GH-regulatory peptides (i.e. TRH, PACAP), in addition to GHRH, were apparently involved [19], showing a contrasting response to what has been reported in mammals [55]. Thus, we hypothesized that during vertebrate evolution the various GH regulatory peptides may have a distinct participation, and exert differential roles, in controlling the mechanisms implicated in pituitary GH synthesis and secretion.

A wide variety of important physiological actions (some being species-specific) in vertebrates are controlled by GH, which shows a pulsatile secretion pattern in several species [44]. It is recognized that, depending on the vertebrate group, a multiplicity of diverse signals, including hypothalamic neuropeptides, participate in the fine regulation of GH synthesis and pulsatile secretion, in order to adapt to specific situations, such as developmental stage, feeding conditions, environmental temperature, or seasonal photoperiod, among others [20]. Data collected from fish to mammals suggest that these regulatory mechanisms involved an ampler diversity of participants in ancient vertebrates and then became more specialized along vertebrate evolution ([20,21,56]).

To investigate the actions of these GH-regulatory peptides among different vertebrate groups, in this study we analyzed and compared the effects of GHRH, PACAP, TRH, GnRH, ghrelin, and SST, upon *gh* mRNA expression and GH release in an *ex-vivo* system employing cultures of pituitary fragments obtained from mammals (rat), birds (chicken), and reptiles (iguana). We also examined the effects of the regulatory peptides upon *Pou1f1* mRNA expression (whose protein product is the transcription factor Pit-1, that mediates GH synthesis) in these experimental models. In addition, an *in-silico* analysis to explore the structure of the corresponding promoters in the *gh* gene was performed, looking for similarities or differences in the number and location of Pit-1 binding sites. Our results offer original information about GH regulatory mechanisms in reptiles, a group poorly studied, and contribute to the analysis about the evolution of these processes in vertebrates.

## 2 Material and methods

### 2.1 Animals

Prepubertal (6 weeks-old) male Wistar rats (*Rattus norvegicus*), weighing around 150 g, were obtained from the animal facilities of the Institute of Neurobiology, UNAM. Animals were housed individually under controlled temperature (22 °C) and lighting (12:12 h light/dark cycle) conditions, with food and water provided *ad libitum*. Pathogen-free fertilized chicken eggs (*Gallus gallus domesticus*, White Leghorn) were kindly donated by Pilgrim's Pride (Querétaro, MX) and were incubated at 39 °C in a humidified air chamber (IAMEX, Mexico City, MX) in the Institute's vivarium. The eggs were rotated one-quarter of a revolution every 50 min until hatching. Male chickens were reared until reaching 4 weeks-old (approximately 800 g) with access to feed and water *ad libitum*, under natural conditions. Juvenile (8-10 months-old, around 150 g) green iguanas (*Iguana iguana*) were obtained from Environmental Management Units (EMU) located in Mexico City and in Guerrero, MX. The animals were maintained in 3 x 3 × 2 m enclosures at 30–35 °C, with a relative humidity of 60–70 %, and a controlled photoperiod of 12:12 h light/dark cycle. They had access to feed (fresh vegetables) and water *ad libitum*. All animals were euthanized by rapid decapitation, following protocol 32, approved by the Institute of Neurobiology Bioethics Committee. Because it is difficult to sex iguanas at this developmental stage, we used both males and females in the experiments.

### 2.2 Peptides

For treatments, the following recombinant peptides (corresponding to the mature sequence) were used: human GHRH (Sigma-Aldrich, St. Louis, MO, USA); mammalian PACAP (Abbiotec, San Diego, CA, USA); rat ghrelin (Abbiotec, San Diego, CA, USA); human GnRH (Sigma-Aldrich, St. Louis, MO., USA); as well as the synthetic peptides TRH and SST (both from Sigma-Aldrich, St. Louis, MO, USA). All peptides were dissolved in high-glucose Dulbecco's Modified Eagle Medium (DMEM, Gibco, Life Technologies, Carlsbad, CA, USA).

**Fig. 1:**
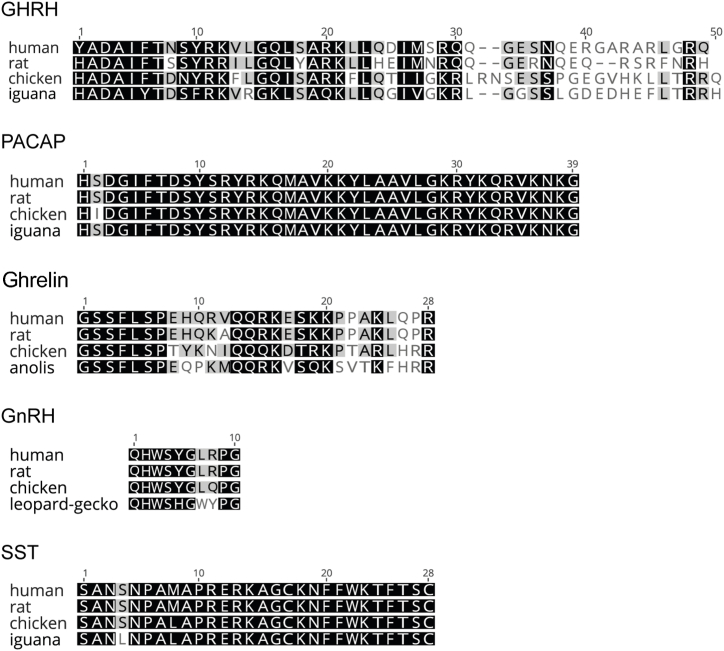
**Multiple alignments of hypothalamic peptides of human, rat, chicken and reptiles.** Similar residues are colored according to BLOSUM62 as follows: black = 100 % similar; dark gray = 80–99 % similar; light gray = 60–79 % similar; white = less than 60 % similar. Since there are no reported sequences for iguana ghrelin and GnRH, those of anole and leopard-gecko, lizards belonging to the order Squamata, were employed for comparison.

Initially, we compared the reported primary structure of these peptides in reptiles, birds and mammals, and confirmed that they are highly conserved among vertebrates (Fig. 1), suggesting that the mammalian sequences might cross-react in the other species and have analogous bioactivity as that of the endogenous peptides. Moreover, it has been shown that several mammalian GH-regulatory peptides exert effects *in vitro*, in non-mammalian vertebrates ([27,57,58]). For these reasons, and since some of the avian and reptilian peptides are not commercially available, we decided to use the mammalian peptides in all experiments.

### 2.3 Experimental design and *ex-vivo* cultures of pituitary fragments

Animals were randomly distributed into control and treatments groups, each containing 4 individuals. For each condition tested, at least two independent experiments were performed for rats and chicken cultures, and one for iguanas (due to the scarcity of the animals). After animal decapitation, each pituitary was immediately collected and washed in a 1.5 ml microcentrifuge tube containing Hank's medium (Gibco, Life Technologies, Carlsbad, CA, USA) supplemented with antibiotics (10,000 units/ml of penicillin and streptomycin, respectively, and 25 μg/ml antifungal Fungizone®, Gibco, Life Technologies, Carlsbad, CA, USA). After washing, each pituitary was cut into 4 fragments and these were placed, together, in a 1.5 ml microcentrifuge tube and stabilized in high-glucose DMEM (Gibco, Life Technologies, Carlsbad,CA, USA) culture medium (100 μl/mg weight) with antibiotics, for 30 min at 37 °C under an atmosphere of 95 % air – 5 % CO_2_. After stabilization, the culture media were replaced with media containing 10 nM of the respective peptide and incubated in the microcentrifuge tubes with the pituitary fragments for either one or 4 h (for GH release or *gh* mRNA expression analysis), at 37 °C. The cultures incubated for 1 h were manually agitated every 20 min, and those incubated for 4 h were gently shaken every hour. Preliminary experiments were performed to determine the final conditions of incubation (i.e. temperature, time, and peptide concentration) as described in the Results section. In the case of *Pou1f1* mRNA expression, the cultures were incubated for 30 min. At the end of the experiments, the culture media were separated from the pituitary fragments, and both were snap-frozen until further analysis.

### 2.4 *In vitro* cultures of dispersed rat pituitary cells

Primary rat pituitary cell cultures were prepared as previously described [59]. Anterior pituitary glands from 20 rats were immediately dissected after decapitation, placed in a sterile 10 cm Petri dish containing 3 ml of sterile Hank's balanced salt solution, Ca^2+^ and Mg^2+^ free (Gibco, Life Technologies, Carlsbad, CA, USA) with antibiotics (10,000 units/ml of penicillin and streptomycin, respectively, and 25 μg/ml antifungal Fungizone®, Gibco, Life Technologies, Carlsbad, CA, USA), washed three times in the same medium, and cut into small fragments (less than 2 mm^3^). Then, these were transferred to a sterile 15 ml tube with DMEM medium (Gibco, Life Technologies, Carlsbad, CA, USA) containing 0.35 % type II collagenase (Worthington, Freehold, NJ, USA), and incubated at 37 °C under a 95 % air – 5 % CO_2_ atmosphere, with agitation and periodic mechanical trituration with a sterile Pasteur pipette, during 15 min to induce tissue disaggregation. The collagenase was inactivated by adding DMEM medium with 10 % fetal bovine serum (FBS). Following dispersion, cells were washed, centrifuged at 1000 rpm for 5 min at 4 °C, the supernatant removed by aspiration and cells were resuspended and counted using a hemocytometer. Then, they were placed (150 x 10^3^ cells/well) in a 24-well plate (Costar, New York, NY, USA) and cultured in a humidified chamber at 37 °C under an atmosphere of 95 % air – 5 % CO_2_. After stabilization, the cells were washed once with fresh DMEM media and then treated for either 1h or 4 h with the test agents (10 nM GHRH, 10 nM ghrelin, or vehicle).

### 2.5 Quantification of GH by ELISA

To analyze GH release, we determined its concentration in samples of culture media from rat, chicken and iguana pituitaries. For chicken and iguana GH, an indirect ELISA assay was performed using our custom-made anti-chicken GH primary antibody at 1:100,000 dilution, as described previously [59], which also cross-reacts with the iguana GH [53]. To quantify the rat GH, a primary anti-rat GH antibody (Millipore, Billerica, MA, USA) was used at 1:40,000 dilution, following the protocol described [60]. In all cases, a secondary HRP-conjugated anti-rabbit IgG antibody (Invitrogen, Carlsbad, CA, USA) was employed at 1:3000 dilution. The signal was developed using ABTS substrate (Roche, Porterville, CA, USA) and it was analyzed in a microplate reader (Bio-Rad, Pleasenton, CA, USA) at 450 nm. The concentration of GH in media was calculated by estimating the maximum binding (B/B0) in the corresponding standard curves, and data are shown as relative proportion (%) of GH concentration in treatments groups in comparison to the corresponding control (treated with vehicle, 100 %).

### 2.6 mRNA extraction and RT-qPCR

Total RNA was extracted from rat, chicken or iguana pituitaries using the TRIzol Reagent (Invitrogen, Life Technologies, Carlsbad, CA, USA), purified using the Direct-zol RNA Mini Prep kit (Zymo Research, Irvine, CA, USA), following the manufacturer's instructions, and then treated with 20 IU of DNase I (Promega, Madison, WI, USA) for 30 min at 37 °C. Subsequently, the corresponding cDNAs were synthesized using 100 U of MLV-V reverse transcriptase (Promega, Madison, WI, USA) in the presence of ribonuclease inhibitor (Thermo Scientific, Waltham, MA, USA), 0.5 μg oligo-d(T), 0.5 μg random hexamers (Invitrogen, Waltham, MA, USA)

and 1 mM dNTPs (Thermo Fisher Scientific, Waltham, MA, USA) for 50 min at 42 °C, followed by 15 min at 70 °C. Later, quantitative real-time PCR (qPCR) was performed using a StepOne System machine (Applied Biosystems, Foster, CA, USA) using SYBR Green reagent (Maxima; Thermo Fisher Scientific, Waltham, MA, USA) in a 10 μl reaction, containing 3 μl cDNA (1:5 dilution) and 0.5 μM of each specific oligonucleotide (Table 1), which were previously validated ([53,60,61]). Changes in the gene expression were analyzed using the comparative threshold cycle (CT) method using formula 2^−ΔΔCT^ [62], where the GH expression was normalized to the RNA levels of the 18S ribosomal reference gene [63]. Results are shown as -fold change of treatment groups as compared to the corresponding controls (1-fold).

**Table 1:** Oligonucleotide primer sequences used in this study.

Oligonucleotide	Sequence (5′- 3′)
**rq18Sf**	TTCAGCACATCCTGCGAGTA
**rq18Sr**	TTGGTGAGGTCAATGTCTGC
**rqGHf**	CTGGCTGCTGACACCTACAAA
**rqGHr**	CAGGAGAGCAGCCCATAGTTT
**rqPou1f**	CATGACTCAGGGTGTGGTCT
**rqPou1r**	CCACGTGATGTCCACAGCGA
**cq18Sf**	CTCTTTCTCGATTCCGTGGGT
**cq18Sr**	TTAGCATGCCAGAGTCTCGT
**cqGHf**	CGCACCTATATTCCGGAGGAC
**cqGHr**	GGCAGCTCCATGTCTGACT
**cqPou1f**	GTCCATGAACAGCAAGCAGG
**cqPou1r**	ACGGATGGGTTTTCTGCGAT
**acq18Sf**	CGCAAATTACCCACTCCCGACC
**acq18Sr**	CCAGACTTGCCCTCCAATGGAT
**giqGHf**	AGAAGTTTGAATCCAACCTCCG
**giqGHr**	AGATATGTCTCCACCTTGTGC
**acqPou1f**	AGGCTGCATATGGGGTGATG
**acqPou1r**	CCAAGGATGGATGGGTGCAT

r = rat; c = chicken; ac = *Anolis carolinensis*; gi = green iguana.

### 2.7 Identification of Pit-1 consensus sites in GH promoters

The Pit-1 sequences of rat, chicken and anole -a lizard closely-related to the green iguana, both belonging to the Squamata order- (GeneBank accession numbers NP_037140.2, NP_001383031.1, and XP_016848011.1, respectively) were aligned using the Muscle algorithm in the Geneious Prime software. DNA-binding domain was predicted based on the rat Pit-1 features annotated in the Protein database (https://www.ncbi.nlm.nih.gov/protein/NP_037140.2?feature=any). To predict the Pit-1 binding consensus sites, we analyzed the promoters of the corresponding GH genes (∼1500 bp upstream of transcription start site) of rat (ID: 24391), chicken (ID: 378781), and anole (ID: 100555670), using the FIMO algorithm (https://meme-suite.org/meme/meme_5.3.2/tools/fimo) of the Motif-Based Sequence Analysis Tools (MEME Suite) and the matrix profile of Pit-1 (POU1F1.p2) from the SwissRegulon Portal (https://swissregulon.unibas.ch/wm/?wm=POU1F1.p2&org=mm9) was used. The binding sites matching with p value < 0.001 were considered as positive.

### 2.8 Statistical analysis

All data are expressed as the mean ± standard error of the mean (SEM). Data were analyzed by Student's independent *t*-test or by one-way analysis of variance (ANOVA), followed by Tukey's multiple comparison test using Prism9 (GraphPad, San Diego, CA, USA). The statistical analyses of gene expression were conducted in log10-transformed values derived from the 2^−ΔΔCT^ normalization. A p value ≤ 0.05 was considered statistically significant.

## 3 Results

### 3.1 Determination of incubation conditions for *ex-vivo* cultures of pituitary fragments

**Fig. 2:**
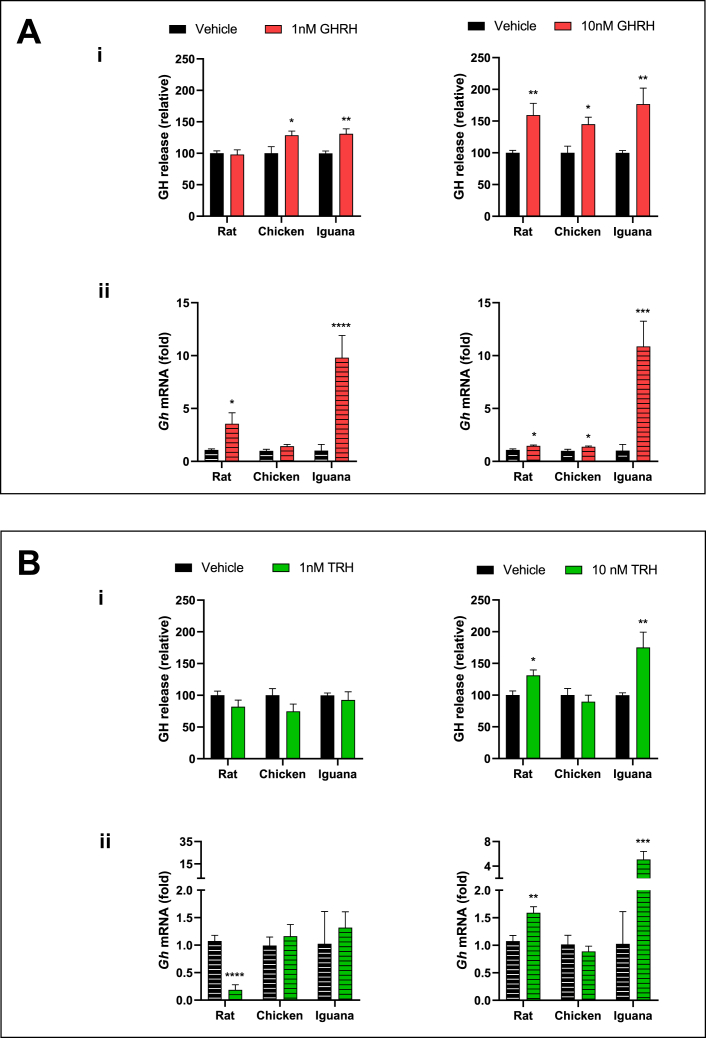
**Effects of GHRH or TRH upon GH secretion and *gh* mRNA expression as a function of dose.***Ex-vivo* cultures of pituitary fragments from rat, chicken or iguana were incubated with 1 or 10 nM of either GHRH (**Panel A**) or TRH (**Panel B**) for 1 h, and their effects were evaluated on **i)** GH secretion, determined by ELISA, and **ii)***gh* mRNA expression determined by qPCR. The bars represent the mean ± SEM, n = 4. *p < 0.05, **p < 0.01, ***p < 0.005, ****p < 0.001 in Student's independent *t*-test. Illustration of results are given as relative proportion (%) of change in the treatment groups in comparison with the controls (100 %) for GH release; while for *gh* mRNA expression they are shown as -fold change as compared to the corresponding controls (1-fold).

**Fig. 3:**
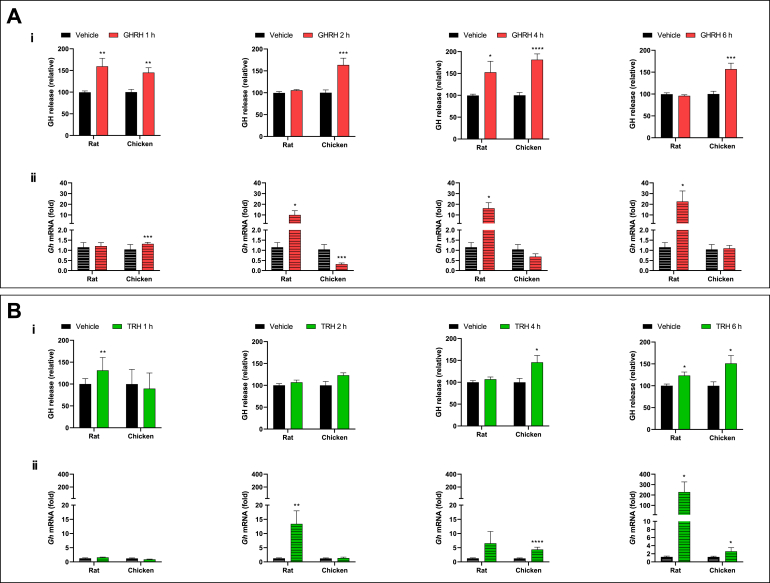
**Effects of GHRH or TRH upon secretion and *gh* mRNA expression as a function of incubation time.***Ex-vivo* cultures of pituitary fragments from rat or chicken were incubated with 10 nM of either GHRH (**Panel A**) or TRH (**Panel B**) for 1, 2, 4, and 6 h, and their effects were evaluated on **i)** GH secretion, determined by ELISA, and **ii)***gh* mRNA expression determined by qPCR. The bars represent the mean ± SEM, n = 4. *p < 0.05, **p < 0.01, ***p < 0.005, ****p < 0.001 in Student's independent *t*-test. Illustration of results are given as relative proportion (%) of change in the treatment groups in comparison with the controls (100 %) for GH release; while for *gh* mRNA expression they are shown as -fold change as compared to the corresponding controls (1-fold).

Firstly, we conducted preliminary studies to determine the conditions (temperature, peptide concentration, time of incubation) that were to be used in the experiments designed to compare the effects of the distinct GH-regulatory peptides on the pituitary cultures in the three vertebrate models employed. It was found that chicken pituitaries cultured at 37 or 39 °C, or iguana pituitaries cultured at 35 or 37 °C, showed no statistical differences in the significant stimulatory effect exerted by GHRH upon *gh* mRNA expression and GH release (Table 2). The concentration of the peptides was chosen after testing the effects of 1 nM and 10 nM GHRH and TRH upon GH regulation after 1 h of incubation (Fig. 2). For GHRH, results showed that the 10 nM dose was consistently effective in significantly increasing GH release and *gh* mRNA expression in cultures from the three species. In comparison, at the 1 nM dose, GH release was stimulated only in chicken and iguana, although to a lesser extent than at the higher dose (Fig. 2A). In turn, 10 nM TRH significantly increased GH secretion and *gh* mRNA expression in rat and iguana, but not in the chicken; whereas the 1 nM dose showed no effects on hormone release in any species and had differential effects upon *gh* mRNA expression: significantly decreased it in the rat, had no effect on chicken, and increased it in the iguana (Fig. 2B). The time-dependent response was assessed in rats and chicken pituitary cultures (iguana pituitaries were not included in this case due to the limited availability of specimens), by testing the application of treatments (10 nM GHRH or TRH) during 1, 2, 4 or 6 h (Fig. 3). GHRH significantly stimulated GH release in both species at 1 h and 4 h of incubation, and higher responses in *gh* mRNA expression at 4h and 6 h of treatment (Fig. 3A). Exposure to TRH showed varying effects along incubation time, and significantly increased GH release and *gh* mRNA expression in both species after 6 h (Fig. 3B). Based on these experiments, the conditions established were the following: incubation temperature: 37 °C; peptide concentration: 10 mM; incubation times: 1 h for GH release and 4 h for *gh* mRNA expression.

**Table 2:** Comparative effects of GHRH treatment upon GH secretion and *gh* mRNA expression in ex-vivo cultures of pituitary fragments at different incubation temperatures.

	GH secretion		*gh* mRNA expression	
**Chicken Pituitaries**	**Control** (ng/ml) Mean ± SEM	**GHRH** (ng/ml) Mean ± SEM	**Control** GH/18S mRNA (fold)	**GHRH** GH/18S mRNA (fold)
**37 °C**	200.2 ± 25.03	318.6 ± 22.91**	1 ± 0.07	1.55 ± 0.14*
**39 °C**	187.3 ± 9.82	266.0 ± 13.48***	1 ± 0.33	1.92 ± 0.55
**Iguana Pituitaries**	**Control** (ng/ml) Mean ± SEM	**GHRH** (ng/ml) Mean ± SEM	**Control** GH/18S mRNA (fold)	**GHRH** GH/18S mRNA (fold)
**35 °C**	13.04 ± 1.51	25.93 ± 3.41**	1 ± 0.30	6 ± 3.61
**37 °C**	12.06 ± 1.47	26.74 ± 4.18**	1 ± 0.44	5.5 ± 2.39

### 3.2 Differential effects of neuropeptides upon regulation of GH release in rat, chicken, and iguana pituitary cultures

**Fig. 4:**
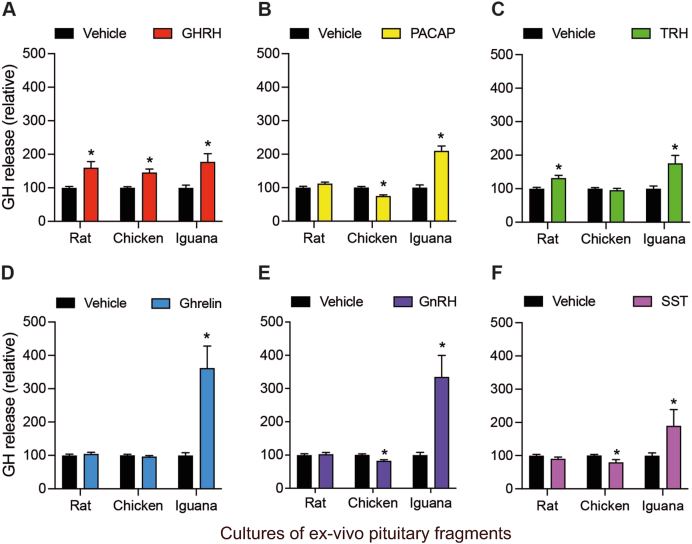
**Effects of peptide-dependent GH release in rat, chicken and iguana pituitary cultures after 1 h of treatment.***Ex-vivo* cultures of pituitary fragments were stabilized by 30 min and then exposed to GHRH, PACAP, TRH, ghrelin, GnRH or SST (10 nM) for 1 h. The GH release was quantified in the culture media using a homologous (rat and chicken) or heterologous (iguana, using an antibody against chicken GH) ELISA. **A.** GHRH induced GH release in rat (*p* = 0.0004), chicken (*p* = 0.0004) and iguana (*p* = 0.0045) pituitary cultures. **B.** PACAP treatment decreased GH release in chicken (*p* = 0.0008), while induced it in iguana (*p* < 0.0001) pituitaries. **C.** TRH caused GH release in rat (*p* = 0.0008) and iguana (*p* = 0.0023) pituitary cultures. **D.** ghrelin induced GH release only in iguana pituitaries (*p* < 0.0001). **E.** GnRH decreased GH release in chicken pituitary cultures (*p* = 0.0137), while in iguana it had a stimulatory effect (*p* < 0.0001). **F.** The SST treatment decreased GH concentration in chicken pituitary culture media (*p* = 0.0053), while it increased in iguana cultures (*p* = 0.0005). Bars represent the mean ± SEM. Asterisks indicate statistically significant differences (Student's independent *t*-test, p < 0.05, n = 4) comparing vehicle vs. treatment within rat, chicken or iguana pituitary cultures.

To compare the effects of GHRH, PACAP, TRH, ghrelin, GnRH, and SST on GH secretion between different vertebrate groups, we analyzed the concentration of GH released to the media of the corresponding cultures of pituitary fragments incubated with the distinct peptides (10 nM) for 1 h (Fig. 4). Results showed that GHRH consistently increased GH release in the three species: rat (159.5 ± 18.5 %), chicken (145 ± 11.1 %), and iguana (176 ± 25.3 %), after 1 h of treatment in comparison to the vehicle controls (Fig. 4A). Interestingly, incubation with the other peptides showed differential effects between species. In rat pituitaries, the GH release was also stimulated by TRH (131.2 ± 8.5 %, Fig. 4C), while PACAP (Fig. 4B), ghrelin (Fig. 4D), GnRH (Fig. 4E), and SST (Fig. 4F)

showed no change in relation to the controls (100 %). On the other hand, in chicken pituitary cultures, the GH release was significantly lowered by PACAP (74.7 ± 4.2 %, Fig. 4B), GnRH (82.7 ± 3.6 %, Fig. 4E), and SST (80 ± 8.3 %, Fig. 4F) treatments, whereas TRH and ghrelin had no effect (Fig. 4C and D). Remarkably, GH secretion from iguana pituitary cultures was strongly stimulated by all peptides: PACAP (209 ± 14.9 %, Fig. 4B), TRH (175 ± 24 %, Fig. 4C), ghrelin (361 ± 66 %, Fig. 4D), GnRH (334 ± 65.1 %, Fig. 4E), and SST (257 ± 53.4 %, Fig. 4F).

**Fig. 5:**
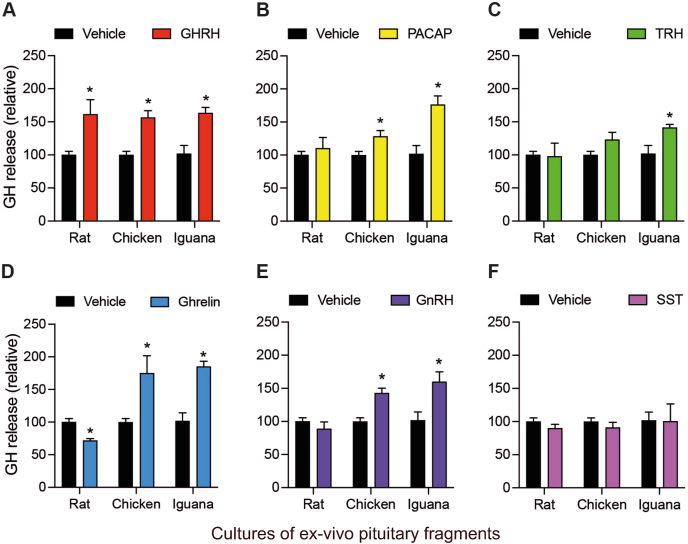
**Effects of peptide-dependent GH release in rat, chicken and iguana pituitary cultures after 4 h of treatment.***Ex-vivo* cultures of pituitary fragments of rat, chicken and iguana were treated as described in Fig. 4, and GH release was quantified by ELISA after 4 h of treatment. **A.** GHRH induced GH release in rat (*p* = 0.0018), chicken (*p* < 0.0001) and iguana (*p* = 0.0023) pituitary cultures. **B.** PACAP treatment increased GH release in chicken (*p* = 0.01) and iguana (*p* = 0.0015) pituitaries. **C.** TRH stimulated GH release in chicken (*p* = 0.0036) and iguana (*p* = 0.018) pituitary cultures. **D.** ghrelin treatment decreased GH release in rat (*p* = 0.024), while induced it in chicken (*p* = 0.0037) and iguana (*p* = 0.0002) pituitaries. **E.** GnRH induced GH release in chicken (*p* = 0.0001) and iguana (*p* = 0.017) pituitary cultures. **F.** SST had no effect on GH release in any species. Bars represent the mean ± SEM. Asterisks indicate statistically significant differences (Student's independent *t*-test, p < 0.05, n = 4) comparing vehicle vs. treatment within rat, chicken or iguana pituitary cultures.

We then analyzed the effects of incubating the corresponding pituitary cultures with the peptides (10 nM) on GH release after 4 h of treatment (Fig. 5). Again, GHRH had a significant stimulatory effect on GH secretion in the three studied species (161 ± 22 %, 156 ± 10.1 %, and 163 ± 8.4 %, in rat, chicken and iguana, respectively; Fig. 5A). Results also showed that rat GH release was not stimulated by any other peptide and was even lowered by ghrelin (71.7 ± 2.9 %, Fig. 5D) at this time. In contrast, at 4 h post-treatment, the chicken GH release was stimulated by PACAP (128.2 ± 8.9 %, Fig. 5B), TRH (145.5 ± 15.7 %, Fig. 5C), ghrelin (175.2 ± 26.5 %, Fig. 5D), and GnRH (142.9 ± 7.4 %, Fig. 5E). On the other hand, as shown in Fig. 5B – E, in iguana pituitary cultures GH release was significantly increased by PACAP (176.3 ± 13.1 %), TRH (141.3 ± 4.7 %), ghrelin (185.1 ± 8.1 %), and GnRH (159.9 ± 15 %). Somatostatin, however, had no effects on basal GH secretion at this incubation time in any species (Fig. 5F).

**Fig. 6:**
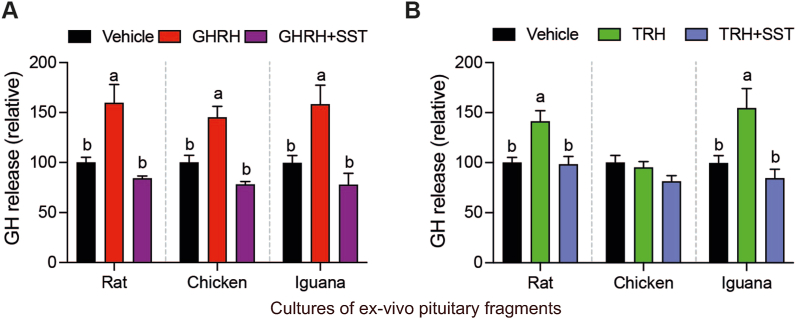
**Effects of SST upon GHRH-stimulated or TRH-stimulated GH release in rat, chicken and iguana pituitary cultures.***Ex-vivo* cultures of pituitary fragments were stabilized for 30 min and then co-incubated with 10 nM GHRH/SST or TRH/SST for 1 h. GH concentration in the culture media was quantified by ELISA, as described in Fig. 4. **A.** Somatostatin inhibited GHRH-stimulated GH secretion in pituitary explants of the three studied species (rat: *F*_(2,21)_ = 6.36, *p* = 0.0069; chicken; *F*_(2,27)_ = 8.34, *p* = 0.0015; iguana *F*_(2,21)_ = 6.36, *p* = 0.0037; one-way ANOVA followed by Tukey's post-hoc test; n = 4). **B.** Somatostatin inhibited TRH-stimulated GH release in rat and iguana pituitary cultures (rat: *F*_(2,17)_ = 8.073, *p* = 0.0034; iguana *F*_(2,26)_ = 6.66, *p* = 0.0046; one-way ANOVA followed by Tukey's post-hoc test; n = 4). Bars represent the mean ± SEM. Letters indicate statistically significant differences (*p* < 0.05) between treatments within pituitary cultures of each species.

The effects of SST upon GHRH-stimulated or TRH-stimulated GH release after 1 h on rat, chicken or iguana pituitary cultures were then investigated (Fig. 6). Results showed that co-incubation with GHRH + SST completely blocked the GHRH-stimulated GH release in the three species (Fig. 6A). Likewise, the TRH + SST co-treatment returned the GH concentration in the culture media to control levels, in comparison with TRH alone, both in rat and iguana pituitaries (Fig. 6B).

### 3.3 Differential effects of peptides upon regulation of *gh* mRNA expression in rat, chicken and iguana pituitary cultures

**Fig. 7:**
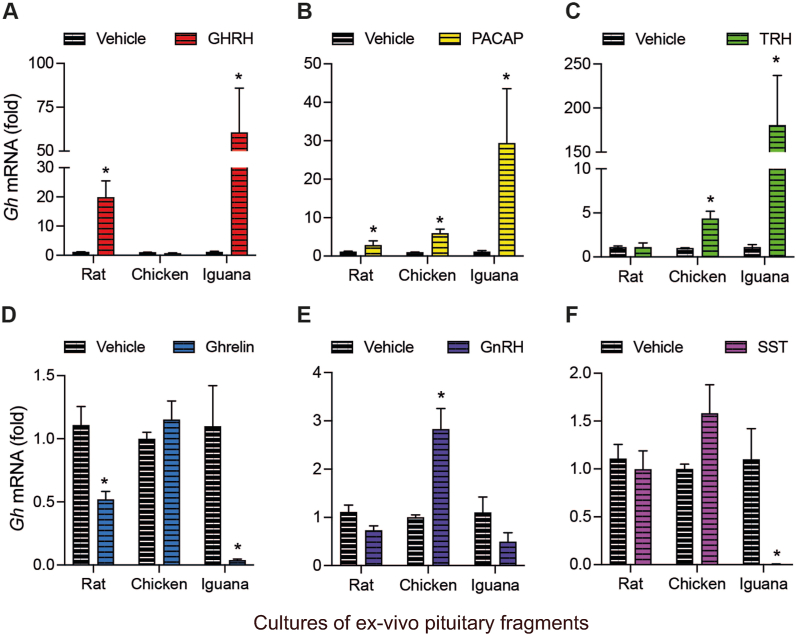
**Effects of peptide treatments on *gh* mRNA expression in rat, chicken and iguana pituitary cultures after 4 h of treatment.***Ex-vivo* cultures of pituitary fragments were stabilized for 30 min, exposed to 10 nM GHRH, PACAP, TRH, ghrelin, GnRH, or SST for 4 h; tissues were then harvested and RNA isolated. The *gh* mRNA expression was quantified by RT-qPCR. **A.** GHRH induced *gh* mRNA synthesis in rat (*p* < 0.0001) and iguana (*p* = 0.015) pituitary cultures. **B.** PACAP induced the rat (*p* = 0.042), chicken (*p* < 0.0001) and iguana (*p* = 0.0005) *gh* mRNAs expression. **C.** Treatment with TRH was effective in inducing *gh* mRNA expression in chicken (*p* < 0.0001) and iguana (*p* < 0.0001) pituitaries. **D.** ghrelin caused a decrease in levels of rat (*p* = 0.019) and iguana (*p* < 0.0001) *gh* mRNA expression. **E.** GnRH treatment induced *gh* mRNA expression only in chicken pituitary cultures (*p* = 0.0005). **F.** SST treatment strongly reduced the *gh* mRNA expression levels in iguana pituitaries (*p* = 0.0012). Bars represent the mean ± SEM. Asterisks indicate statistically significant differences (Student's independent t-test, p < 0.05, n = 4) comparing vehicle vs. treatment within rat, chicken or iguana pituitary cultures.

Quantification of the *gh* mRNA expression levels was determined in *ex-vivo* cultures of pituitary fragments from the three species after 4 h of treatment with the different peptides. Results showed that, in comparison to the corresponding control (1-fold), expression of rat *gh* mRNA was induced strongly by GHRH (19.8 ± 4.1-fold, Fig. 7A) and by PACAP (2.8 ± 1.1-fold, Fig. 7B); in contrast, ghrelin decreased it (0.47 ± 5.9-fold, Fig. 7D), while TRH, GnRH and SST had no effect. On the other hand, it was found that expression of chicken *gh* mRNA was significantly increased by PACAP (5.8 ± 1.1-fold, Fig. 7B), TRH (4.3 ± 0.8-fold, Fig. 7C), and GnRH (2.8 ± 0.42-fold, Fig. 7E), whereas GHRH, ghrelin, and SST showed no significant effects (Fig. 7A, 7D, 7F). Interestingly, the iguana pituitaries exhibited the most striking changes in response to treatments with the distinct peptides in comparison with responses observed in rat and chicken. Results indicated that iguana *gh* mRNA expression was strongly induced by GHRH (60 ± 25.3-fold, Fig. 7A), PACAP (29 ± 14.2-fold, Fig. 7B), and TRH (180 ± 56.5-fold, Fig. 7C), while treatments with ghrelin and SST intensely reduced it (0.04 ± 0.9-fold and 0.01 ± 0.1-fold, respectively; Fig. 7D and F). Instead, GnRH showed no significant effect.

### 3.4 GHRH and ghrelin effects on GH release and *gh* mRNA expression in dispersed rat pituitary cells cultures

**Fig. 8:**
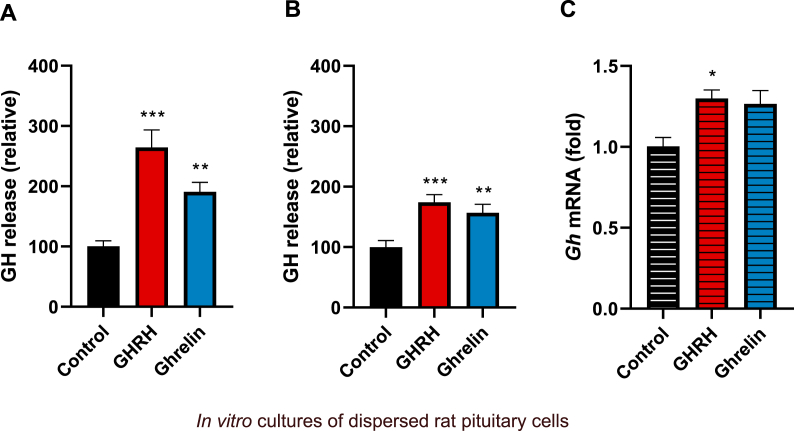
**Effects of GHRH and ghrelin upon GH secretion and *gh* mRNA expression in rat pituitary dispersed cell cultures.** Primary rat pituitary cell cultures were incubated with 10 nM GHRH or ghrelin for 1 h and 4 h. **a)** GHRH (p = 0.0001) and ghrelin (p = 0.0002) induced GH release after 1 h of incubation. **b)** GHRH (p = 0.0002) and ghrelin (p = 0.0043) induced GH release after 4 h of incubation. **c)** GHRH (p = 0.0175) and ghrelin (p = 0.0576) induced *gh* mRNA expression after 4 h of incubation. Bars represent the mean ± SEM. Asterisks indicate statistically significant differences (Student's independent *t*-test, p < 0.05, n = 5).

Fig. 8 shows that in primary cultures of dispersed rat pituitary cells, GHRH induced a significant increase in GH secretion both at 1 h (264.6 ± 29 %, Figs. 8A) and 4 h (174.3 ± 12.5 %, Fig. 8B) of incubation in comparison to their corresponding controls (100.7 ± 9.2 % and 100 ± 10.7 %). Likewise, in this culture model, ghrelin was also able to significantly induce GH release at both times (190.7 ± 15.8 % and 156.8 ± 14.2 %, respectively; Fig. 8A and B), although to a lesser extent than GHRH. On the other hand, Fig. 8C shows that after 4 h of incubation, the *gh* mRNA expression was significantly augmented by GHRH (1.29 ± 0.05-fold) whereas ghrelin showed a tendency to increase it (1.26 ± 0.08-fold) but was not statistically significant (p = 0.0576).

### 3.5 Effects of peptides on *Pou1f1* mRNA expression in rat, chicken and iguana pituitary cultures

**Fig. 9:**
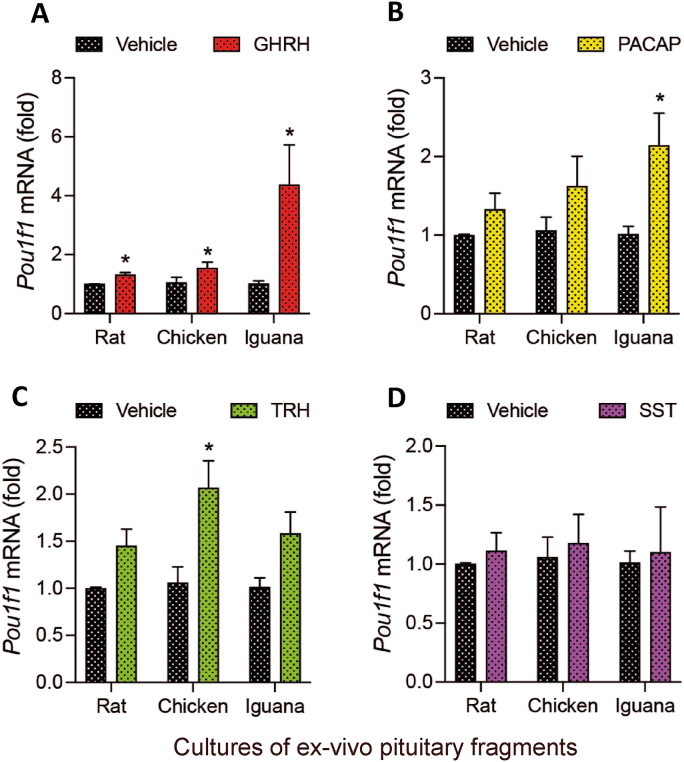
**Effects of the peptides on *Pou1f1* mRNA expression in rat, chicken and iguana pituitary cultures.***Ex-vivo* cultures of pituitary fragments were stabilized for 30 min, and then exposed to either GHRH, PACAP, TRH, or SST for another 30 min. Tissues where then harvested and RNA isolated. The *Pou1f1* mRNA expression was quantified by RT-qPCR. **A.** GHRH stimulated *Pou1f1* mRNA synthesis in rat (*p =* 0.012), chicken (*p =* 0.045) and iguana (*p =* 0.024) pituitary cultures. **B.** PACAP increased *Pou1f1* mRNA levels only in iguana (*p =* 0.02) pituitaries. **C.** TRH only induced the chicken *Pou1f1* mRNA expression (*p =* 0.015). **D.** Treatment with SST did not affect the *Pou1f1* mRNA expression in any species, in comparison with the controls. Bars represent the mean ± SEM. Asterisks indicate statistically significant differences (Student's independent *t*-test, *p* < 0.05, *n =* 4) comparing vehicle vs. treatment within rat, chicken or iguana pituitary cultures.

The transcription factor Pit-1, involved in pituitary GH synthesis, is encoded by the *Pou1f1* gene. Fig. 9 shows the response of *Pou1f1* mRNA expression levels when pituitary cultures were incubated for 30 min with either GHRH, PACAP, TRH, or SST. Results revealed that GHRH significantly stimulated *Pou1f1* mRNA expression in the three species (1.3 ± 0.07; 1.6 ± 0.16; and 4.3 ± 1.3-fold, in rat, chicken and iguana pituitary cultures, respectively), being highest in the reptile (Fig. 9A). In turn, treatment with PACAP was only effective in inducing the expression of iguana *Pou1f1* mRNA (2.1 ± 0.4-fold, Fig. 9B); whereas TRH showed a significant effect only on the expression of the chicken *Pou1f1* mRNA (2 ± 0.2-fold, Fig. 9C). On the other hand, SST had no effects in any of the three species (Fig. 9D). No changes were found in the cultures treated with ghrelin nor GnRH in rat and chicken pituitaries (data not shown).

### 3.6 Analysis of Pit-1 consensus binding sites in rat, chicken and anole *gh* gene promoters

**Fig. 10:**
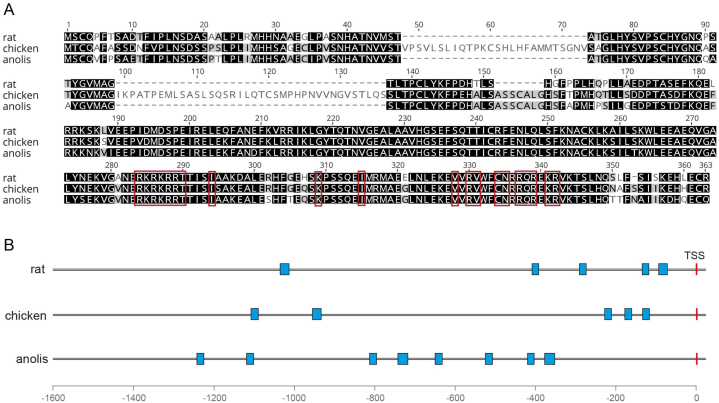
**Comparison of Pit-1 primary structure from mammalian, bird and reptile species, and analysis of Pit-1 consensus binding sites in *gh* gene promoters. A.** Multiple alignment of rat (NP_037140.2), chicken (NP_001383031.1) and anole (XP_016848011.1) Pit-1 amino acid sequences. Similar residues are colored according to BLOSUM62 as follows: black = identical; gray = similar; white = no similarity. Red boxes highlight residues of the DNA-binding domain, which are identical in all three species. **B.** Diagram showing the location of predicted Pit-1 consensus binding sites (blue boxes) in the *gh* gene promoters of rat, chicken and anolis. The red line indicates the transcription start site (TSS). (For interpretation of the references to color in this figure legend, the reader is referred to the Web version of this article.)

To study the role that *gh* gene structure could play on differential peptide-dependent GH regulation between vertebrates, the sequences of *gh* gene promoters of rat, chicken, and anole (a lizard closely-related to the green iguana) were analyzed to identify the location and number of Pit-1 consensus binding sites in each species. Initially, we compared the primary structure of rat, chicken and anole Pit-1s and found that these proteins are highly conserved among vertebrates, particularly at the DNA-binding site, which are 100 % identical between the analyzed species (Fig. 10A). This allowed the assumption that Pit-1 transcription factors in these distinct animals recognize very similar consensus sites in DNA. Then, we looked for the number and location of the Pit-1 consensus binding sites in a region encompassing ∼1500 bp upstream of the transcription start site (TSS) in the respective *gh* promoters. Results of this examination showed that the *gh* gene promoters from both rat and chicken have five Pit-1 consensus sites each (rat: -1012, -400, -284, -131, and -91 bp from TSS; chicken: -1086, -936, -224, -175, and -132 bp from TSS, respectively); while eight Pit-1 consensus sites were predicted in the anole *gh* promoter (-1113, -992, -692, -624, -533, -411, -308, and -266 bp from TSS, Fig. 10B).

## 4 Discussion

Growth hormone plays essential roles in the physiology of vertebrates, not only regulating growth and development, but also modulating important biological processes such as metabolism, immunity, osmoregulation, reproduction and neuroprotection [1], among others. To perform these actions, GH synthesis and release must be finely controlled and regulated at its primary site of synthesis, the pituitary. Several peptides are known to be involved in the regulation of GH and it has been proposed that they may have differential effects and potency along the phylogenetic scale ([20,21,56]). These intricate processes have been studied considerably in mammals but are still poorly understood in other vertebrate groups; in particular, very little is known in reptiles. The available information suggests that the regulation of GH secretion from somatotrophs shows a higher diversity in ancient vertebrates, where a larger variety of neuropeptides are involved ([20,21,52,64]. To contribute to the understanding of how the regulation mechanisms of GH synthesis and release have evolved during vertebrate phylogeny, here we performed a comparative analysis of the effect of several peptides on GH regulation in representative species of reptiles, birds, and mammals.

It has been described that, during evolution of vertebrates, several anatomical connections between the hypothalamus and the pituitary gland can vary, which contribute to a multimodal and multifactorial control of communication [65]. Also, hormone-secreting cell lineages vary their distribution in the anterior pituitary, with some being more distinctly regionalized than others [66]. In the case of somatotrophs, the GH-producing cells, it has been described that they are more concentrated in the caudal lobe of the *pars distalis* in the iguana [53] and in the chicken [61] although some proportion of GH-immunoreactive cells are also found in the cephalic lobe in both species; in contrast, the somatotrophs are more widely distributed in the anterior pituitary of the rat [67]. We decided to perform this study employing cultures of *ex-vivo* pituitary explants from the three species, instead of primary cultures of pituitary dispersed cells. The rationale behind was to try to preserve the tissue's cytoarchitecture in order to keep important cell to cell and cell matrix paracrine/juxtacrine/intracrine interactions in their tridimensional setting and more closely simulate physiological conditions, in contrast to those attained by the use of two-dimensional primary dispersed cells grown *in vitro* [68] where enzymatic treatment disrupts tissue structure and contacts between neighboring cells and may affect some of cell surface components.

Each pituitary was cut into 4 sections to facilitate the penetration of the peptides tested in the distinct treatments, and the four fragments were incubated in the same tube so that the responses obtained corresponded to the whole gland. We initially standardized dose, temperature and time to optimize the culture incubation conditions to be used in the experiments.

To determine the best peptide concentration, we examined GHRH and TRH doses (1 and 10 nM) that were within the physiological interval in the species used, guided by preliminary studies where a wider range (0.1–100 nM) was employed. We observed that, in the case of GHRH, the 10 nM dose was reliably effective to stimulate both GH release and *gh* mRNA expression in the three species, and these findings align well with those previously reported in fish and amphibian pituitaries [24,26]. On the other hand, TRH results showed that the higher dose provoked significant increases in both parameters in rat and iguana, but not in the chicken; whereas at the lower dose it exhibited differential responses in *gh* mRNA expression among the species studied: in the rat it was significantly diminished -a result that coincides with an inhibitory effect previously described [69]- while in contrast it was importantly increased in the iguana, and had no effect in the chicken.

Since the species studied (two endotherms and one ectotherm) naturally have different average body temperatures (chicken: 39 °C; rat: 37 °C; iguana: 35 °C), it was important to determine if there were any changes in the response due to the incubation temperature of the cultures. Thus, an experiment was performed to compare the effects of incubating 10 nM GHRH with chicken pituitaries at 39 °C and 37 °C on one hand, and iguana pituitaries at 35 °C and 37 °C on the other. Results showed that there were no significant differences upon GH release and *gh* mRNA expression when the cultures were incubated at these distinct temperatures.

Pituitary hormone synthesis and secretion are processes that occur over different time frames. While release generally occurs within minutes in response to a secretagogue, regulation of gene expression and protein synthesis could take up to hours. Thus, we explored different treatment times (1, 2, 4, and 6 h) to analyze the corresponding responses to incubation with these secretagogues in rat and chicken pituitary cultures. It was found that GH release was promptly stimulated by GHRH since the first hour, likely because the hormone was already accumulated in secretory granules in the pituitary and only required activation of the phospholipase C pathway [2], whereas *gh* mRNA synthesis took longer times since it requires activation of additional participants such as PKA and CREB transcription factor [70].

Previous studies, mainly performed in primary dispersed pituitary cells cultures, have shown that GHRH stimulates pituitary GH release in mammals [71], birds [29], reptiles [27], amphibians [26], and fish [24]. Our results showing that, from all tested peptides, only GHRH was able to consistently stimulate GH release in all three species (rat, chicken and iguana), support the notion that GHRH is the universal stimulator of GH secretion across vertebrates.

In addition to the clear effect of GHRH in rat pituitary cultures, here we observed that after 1 h of incubation, TRH also stimulated GH release, as described before ([33,72]). This result, however, was not maintained after 4 h of exposure. Neither of the other peptides studied showed any effect on rat pituitary GH secretion at the times tested, except for ghrelin that depressed GH release after 4 h of treatment. Although this finding is controversial to what has been described previously in primary cultures of dispersed pituitary cells in rats ([73,74] or humans [75], it has also been reported that the ghrelin receptor (GHSR) may activate inhibitory G proteins, leading to inhibition of adenylate cyclase activity ([[76], [77], [78], [79], [80], [81]]). This suggests that ghrelin could be involved in inhibitory pathways in *ex-vivo* cultures of rat pituitary fragments. In this regard, the type of experimental model employed may have a strong effect on cellular response to treatments, as reported earlier [82] in goldfish, where it was found that GnRH-stimulated mRNA expression of several pituitary hormones (FSH-β, LH-β, and GH) showed differential time- and dose-related responses in cultured pituitary fragments in comparison to primary dispersed pituitary cells or *in vivo* experiments. Moreover, a clear disparity of results was observed upon GH release in male rats, in experiments that compared the response of pituitary sections versus dissociated cell cultures to GHRH [67]. To explore this possibility, we also analyzed the effect of ghrelin in primary cultures of rat pituitary dispersed cells and found a significant increase of GH release both at 1 h and 4 h of incubation. Undoubtedly, the mechanism involved in this controversial effect, that may depend on the culture model used, deserves further research to better understand the influence of neighboring cell to cell interactions when the tissue cytoarchitecture is more preserved.

The effect of several peptides on GH regulation in avian pituitaries has been previously reported both *in vivo* and *in vitro*. It is known that, in addition to GHRH, GH release is also stimulated in chickens by PACAP [37], TRH ([43,83,84]), and ghrelin [50]. Likewise, we also found that all these peptides had actions upon GH release. In addition, results showed for the first time that GnRH also stimulated GH release in chicken pituitaries at 4 h of incubation, an effect not previously reported in birds. By contrast, we found that after 1 h treatment, the basal rate of GH release was decreased by PACAP and GnRH, in a manner similar to SST. These dual inhibitory and stimulatory effects of GnRH were consistent to those previously reported in rats [85], where it was found that GH secretion in cultures of pituitary cell aggregates decreased transiently after 20–60 min of treatment with GnRH, followed by an increase in secretion above baseline when the peptide was withdrawn; whereas in cultured hemi-pituitaries a significant stimulatory effect of GnRH upon GH release was observed without rebound.

Results showing that all tested peptides were able to induce GH secretion from iguana pituitaries are consistent with the proposal that GH regulation in reptiles involve the participation of more peptides than in mammals. As mentioned before, there are very limited studies on GH regulation in reptiles. Our findings in iguana confirm those of Denver and Licht ([27,57,58]), describing that GHRH and TRH stimulated GH release in hatchling or adult turtles (*Pseudemys scripta elegans* or *Chrysemus picta bellii*) in either whole gland or hemi-pituitaries cultures. Furthermore, here we provide new evidence that PACAP, ghrelin, and GnRH have clear actions as secretagogues in reptiles, since they strongly stimulated GH release at both incubation times tested, with a relative potency even higher that that shown by GHRH or TRH. Intriguingly, we observed that SST also stimulated GH secretion during the most acute treatment. Although it is known that the primary action of SST on GH release is inhibitory in fish and other vertebrates ([20,21,56]), it has also been reported that, under certain conditions (for example, at low doses), SST is able to stimulate GH secretion in pituitary cells from chickens [86], pigs ([87,88]), or baboons [89], via the subtype 5 somatostatin receptor and cAMP signaling activation. It was shown here that such effect is, likewise, also present in iguana. In addition, evidence was obtained that the archetypal inhibitory effect upon GHRH- or TRH-stimulated GH release, previously described in mammals [71], birds [90], and fish [91], is also conserved in reptiles. On the other hand, while it has been described that SST had no effects on *gh* mRNA transcription in mammals ([92,93]) or fish (in primary pituitary cell cultures from orange-spotted grouper [94] or pituitary organ cultures in rainbow trout [95]), our results showed that SST inhibited basal *gh* mRNA expression in iguana. Although it is generally accepted that the main action of SST relates to the inhibition of basal and stimulated GH release, some reports show that it can also affect GH synthesis and *gh* transcription. In this regard, it was described that SST significantly decreased the transcription of *gh* mRNA in rats treated with an antiserum against rat GHRH, or when received a continuous infusion of SST via an atrial cannula in comparison with the control that received vehicle [96]. In addition, it was shown that increased activity of SSTR2 induced an attenuation of GH synthesis in GH3 rat pituitary tumor cells [97] and STTR3 activation mediated transcriptional repression of *gh* in GC pituitary tumor cell line [98]. Interestingly, it was also described that SST was capable to significantly inhibit basal extra-pituitary *gh* mRNA expression in chicken bursal B-lymphocytes cultures [99].

It is known that, besides their role in controlling GH secretion, some of these peptides are also involved in the regulation of *gh* mRNA expression through the activation of the cAMP-PKA signaling pathway ([1,100]). The regulation of *gh* mRNA expression is independent of the regulation of GH release. While secretion is an early event and involves calcium mobilization, GH synthesis is a more delayed response and involves activation of the cAMP pathway. This could explain some of the differences we found between release and expression. For example, GHRH stimulated GH release in chicken pituitaries but had no effect on *gh* mRNA levels, probably because the effect on expression is at a different time. These are novel results since there are very few studies in which the regulation of GH secretion and synthesis is analyzed simultaneously between distinct species. Although similar incubation conditions were employed in the cultures, it should be considered that some differences in maturation stages and sex of the animals used existed (prepubertal males in rats, juvenile males in chickens, juvenile males and females in iguanas). Furthermore, these results clearly show that the peptides tested had a differential impact upon *gh* mRNA expression between the vertebrate groups studied. Interestingly, in iguana cultures of pituitary fragments it was shown for the first time that *gh* mRNA expression was vigorously stimulated by GHRH, TRH, and PACAP, and the response to these peptides was much stronger than that observed in their mammalian or avian counterparts. In contrast, both ghrelin and SST exerted a profound inhibition of *gh* mRNA basal expression. This effect had not been reported in endotherms, but in salmonids it was described that SST inhibited transcription of *gh* ([101,102]). Overall, these findings suggest that the regulatory mechanisms involved in peptide-dependent GH synthesis and release in iguana are more akin to those of fish than to mammals.

Our study shows clear differential *in vitro* responses and potencies among the peptides employed upon GH secretion and *gh* mRNA expression in representative species of three groups of vertebrates (Table 3). Also, it shows that the repertory of regulatory participants in the iguana system is more diverse that that in the rat. These results contribute to support the proposal that GH regulation became more specialized along vertebrate evolution ([20,21,52]).

**Table 3:**
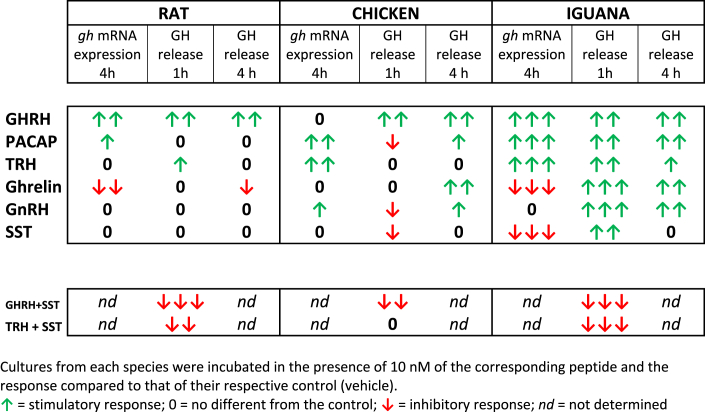
*Effects of several peptides upon gh expression and GH release* in *ex-vivo* cultures of pituitary fragments from different vertebrates.

To investigate the mechanisms underlying these differential responses, we analyzed the expression of *Pou1f1*, an early response gene whose protein product is the transcription factor Pit-1, an output gene of the cAMP/PKA signaling pathway that mediates GH synthesis. It is known that in the peptide-induced signaling pathway, the adenylyl cyclase (AC) is activated after the regulatory peptides bind to their specific membrane receptor ([21,103]). This enzyme catalyzes the formation and accumulation of cyclic adenosine monophosphate (cAMP), which in turn activates the protein kinase A (PKA) [104]. Then, PKA phosphorylates and activates the cAMP-responsible element-binding protein (CREB), which after binding the cAMP response elements (CREs) modulates the cAMP-PKA-dependent gene expression, including the *Pou1f1* gene [103]. Once synthesized, Pit-1 then regulates the expression of the *gh* gene [104].

Our results showed that expression of *Pou1f1* was stimulated with higher potency by GHRH and PACAP in iguana pituitary cultures, in comparison to the response observed in rat and chicken counterparts. This finding suggests that Pit-1 could be involved in the mechanisms underlying the stronger *gh* expression responsiveness observed in the reptile model. Furthermore, the *in-silico* analysis showed that the intrinsic evolutionary changes noted in the structure of *gh* gene promoters could also be implicated in the differential responses observed in this study, since the reptilian *gh* gene promoter contains more Pit-1 binding consensus sites (8) in comparison to chicken and rat (5). It is possible, however, that other mechanisms could be involved to explain the increased sensitivity of the pathway in the iguana pituitary; for example, higher expression of regulatory peptide receptors in somatotrophs; lower rate of desensitization; or higher expression of the genes involved in the pathway. Therefore, further experiments are needed to test these hypotheses.

## 5 Conclusions

Taken together, the results of this study indicate that the peptide-mediated mechanisms that control pituitary *gh* expression and GH release have undergone important differential changes during vertebrate evolution, and that in reptiles a more diverse interaction of these factors is involved in the GH regulatory network, as compared to birds and mammals.

## Funding statement

This work was supported by grants IN201817, IN227020, IN209621, IN215522, IA200622 from Programa de Apoyo a Proyectos de Investigación e Innovación Tecnológica de la Dirección General de Asuntos del Personal Académico, Universidad Nacional Autónoma de México (PAPIIT-DGAPA-UNAM, Mexico City, Mexico), and UNAM grant 1130-202-002 to C.A.; as well as by grants 285004 and 214971 from Consejo Nacional de Ciencia y Tecnología (10.13039/501100003141CONACYT, Mexico City, Mexico). A doctoral fellowship to VAUS (# 787975) was provided by CONACYT.

## Data availability statement

The data that supports the findings of the current study are available from the corresponding author upon reasonable request.

## Ethics declaration

This study was reviewed and approved by the Institute of Neurobiology Bioethics Committee, with the approval number: 32. The experimental work was in alignment with the bioethical norms of the National Health and Medical Research Council.

## CRediT authorship contribution statement

**Valeria A. Urban-Sosa:** Writing – review & editing, Writing – original draft, Methodology, Investigation, Formal analysis, Data curation, Conceptualization. **José Ávila-Mendoza:** Writing – review & editing, Writing – original draft, Validation, Supervision, Methodology, Investigation, Formal analysis, Data curation, Conceptualization. **Martha Carranza:** Methodology, Investigation. **Carlos G. Martínez-Moreno:** Resources, Methodology, Investigation. **Maricela Luna:** Resources, Methodology, Investigation. **Carlos Arámburo:** Writing – review & editing, Writing – original draft, Validation, Supervision, Resources, Project administration, Funding acquisition, Formal analysis, Conceptualization.

## Declaration of competing interest

The authors declare that they have no known competing financial interests or personal relationships that could have appeared to influence the work reported in this paper.
